# Supplementary material for: Splice-Junction-Based Mapping of Alternative Isoforms in the Human Proteome
Source: Cell Rep. Author manuscript; Available in PMC 2020 Jan 15. (PMC6961840; doi:10.1016/j.celrep.2019.11.026)
Supplement: 2 [file NIHMS1546469-supplement-2.zip › DF1/PXD006675/Aorta/Aorta_21_MPST_AGVGAMGGGGAAGPAR.pdf]

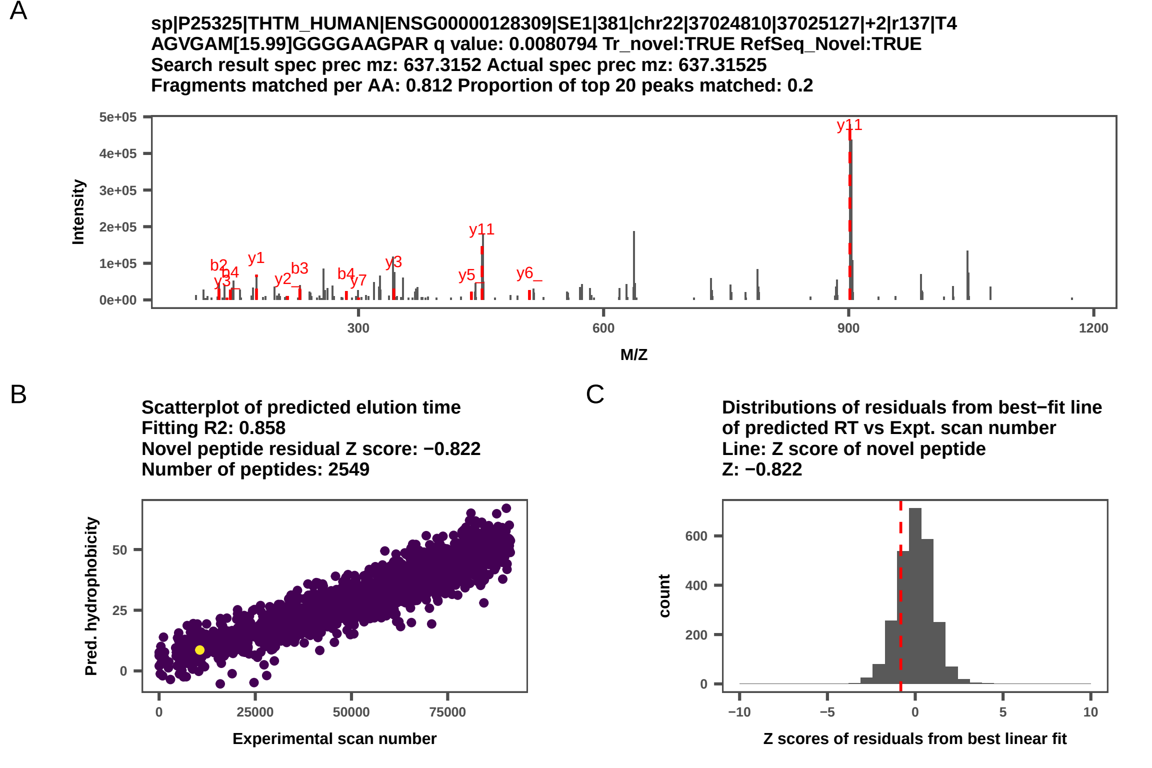
A
sp|P25325|THTM_HUMAN|ENSG00000128309|SE1|381|chr22|37024810|37025127|+2|r137|T4
AGVGAM[15.99]GGGGAAGPAR q value: 0.0080794 Tr_novel:TRUE RefSeq_Novel:TRUE
Search result spec prec mz: 637.3152 Actual spec prec mz: 637.31525
Fragments matched per AA: 0.812 Proportion of top 20 peaks matched: 0.2
Intensity
5e+05
4e+05
3e+05
2e+05
1e+05
0e+00
y11
y11
b2
y1
b3
y3
b4
y3
y2_
b4
y7
y5
y6_
300
600
900
1200
M/Z
B
Scatterplot of predicted elution time
Fitting R2: 0.858
Novel peptide residual Z score: −0.822
Number of peptides: 2549
Pred. hydrophobicity
50
25
0
0
25000
50000
75000
Experimental scan number
C
Distributions of residuals from best−fit line
of predicted RT vs Expt. scan number
Line: Z score of novel peptide
Z: −0.822
count
600
400
200
0
−10
−5
0
5
10
Z scores of residuals from best linear fit
